# Factors influencing emergency medical readmission risk in a UK district general hospital: A prospective study

**DOI:** 10.1186/1471-227X-5-1

**Published:** 2005-01-21

**Authors:** Georgios Lyratzopoulos, Daniel Havely, Islay Gemmell, Gary A Cook

**Affiliations:** 1 Directorate of Clinical Services and Public Health, Norfolk, Suffolk and Cambridgeshire Strategic Health Authority, Fulbourn, UK; 2 Evidence for Population Health Unit, University of Manchester, Manchester, UK; 3 Department of Epidemiology, Stockport NHS Trust, Stockport, UK

## Abstract

**Background:**

Over recent years increased emphasis has been given to performance monitoring of NHS hospitals, including overall number of hospital readmissions, which however are often sub-optimally adjusted for case-mix. We therefore conducted a study to examine the effect of various patient and disease factors on the risk of emergency medical readmission.

**Methods:**

The study setting was a District General Hospital in Greater Manchester and the study period was 4.5-years. All index emergency medical admission during the study period leading to a live discharge were included in the study (n = 20,209). A multivariable proportional hazards modelling was used, based on Hospital Episodes Statistics data, to examine the influence of various baseline factors on readmission risk. Deprivation status was measured with the Townsend deprivation index score. Hazard ratios (HR) and associated 95% confidence intervals (CI) of unplanned emergency medical admission by sex, age group, admission method, diagnostic group, number of coded co-morbidities, length of stay and patient's deprivation status quartile, were calculated.

**Results:**

Significant independent predictors of readmission risk at 12 months were male sex (HR 1.13, CI: 1.07–1.2), age (age >75 (HR 1.57, CI 1.45–1.7), number of coded co-morbidities (HR for >4 coded co-morbidities: 1.49 CI: 1.26–1.76), admission via GP referral (HR 0.93, CI 0.88–0.99) and primary diagnosis of heart failure (HR 1.33, CI: 1.16–1.53) and chronic obstructive pulmonary disease/asthma (HR 1.34, CI: 1.21–1.48). Higher level of deprivation was also significantly and independently associated and with increased emergency medical readmission risk at three (HR for the most deprived quartile 1.21, CI: 1.08–1.35), six (HR 1.21, CI: 1.1–1.33) and twelve months (HR 1.25, CI: 1.16–1.36).

**Conclusions:**

There is a potential for improving health and reducing demand for emergency medical admissions with more effective management of patients with heart failure and chronic obstructive airways disease/asthma. There is also a potential for improving health and reducing demand if reasons for increased readmission risk in more deprived patients are understood. The potential influence of deprivation status on readmission risk should be acknowledged, and NHS performance indicators adjustment for deprivation case-mix would be prudent.

## Background

Over recent years increased emphasis has been given to performance monitoring of NHS hospitals and various quality indicators based on analysis of routine administrative data have been devised[1]. The 2003 dataset of the Commission of Health Improvement indicators includes 28-day hospital readmission rates following any hospital admission, and also readmission rates following admission for stroke and hip fracture[2]. Although indicators are currently standardised for age and sex^1^, they are not adjusted for potential case-mix differentials in disease severity, co-morbidity and patient deprivation status.

Unplanned hospital re-admissions may represent adverse events and could therefore indicate poor quality of care[3,4]. The interpretation of variation in readmission rates between healthcare organisations is nevertheless complicated[5,6]. Broadly a readmission could be due to healthcare factors (e.g. sub-optimal health and social care, either at hospital or within primary/social care structures), patient factors (e.g. poor treatment adherence), disease factors (e.g. natural disease progression), or a combination of all the above. Readmissions due to healthcare and patient factors could be assumed to be potentially avoidable. There is insufficient evidence about the proportion of hospital readmissions that could be judged to be due to healthcare factors, as estimates of the proportion of medical readmissions that are due to healthcare factors vary widely between 9 and 48%[4].

Although current NHS performance indicators use readmission rates at 28-days[1,2], there is lack of consensus in the literature about the choice of optimal time interval, with different studies choosing different intervals, ranging from one week to one year[4]. Intuitively shorter time intervals are more likely to represent "avoidable" readmissions due to poor quality care, nevertheless this may be more applicable in the case of elective surgical admissions rather than emergency medical ones. Moreover, for chronic medical conditions (such as diabetes, chronic obstructive pulmonary disease and heart failure), even a "delayed" readmission may represent a failure of disease management due to deficiencies in healthcare quality[4].

It can be hypothesised that factors such as patient sex, length of stay, number of coded co-morbidities, method of admission (e.g. presentation to the Accidents and Emergency department or GP referral), as well as patient deprivation, independently influence the probability of hospital readmission. Lower socioeconomic status in particular is independently associated with increased risk for many adverse health outcomes[7], including hospital readmission due to conditions such as heart failure[8]. The increasing demand for emergency medical admissions[9] makes epidemiological studies of medical readmissions a priority. Additionally, emergency medical admissions constitute a sizeable proportion of all hospital admissions, and can therefore play an important role in the overall performance of NHS hospitals under the current set of indicators[1,2]. We therefore conducted a study to examine the effect of several patient and disease factors on the risk of emergency medical readmission at various time intervals following an index emergency medical admission.

## Methods

### Context

This work was carried out as part of the routine function of Stockport NHS Trust Clinical Effectiveness Unit, and in relation to work commissioned by the then "Emergency Demand Management Group" of the Stockport Primary Care Trust. The objective was to accurately describe the epidemiology of emergency medical readmissions so that demand management strategies (e.g. appropriate targeting of resources to patients with certain conditions, or certain types of presentation) could be informed.

### Setting

Stockport NHS Trust is a district general hospital in Greater Manchester, serving a reference population of about 300,000. About 85% of all patients emergency medical admissions are from Stockport, a population with a slightly lower, compared to the England and Wales average, Standardised Mortality Ratio from all causes (all ages) of 96 (95% CI 94–98)[10].

### Data source, population and follow-up period

Hospital Episodes Statistics Data from April 1997 to September 2001 for Stockport NHS Trust were analysed and all emergency medical admissions in Stockport residents leading to live discharge were identified. An emergency medical admission was defined as an emergency hospital admission to any medical specialty in person over 18 years of age. Some persons had more than one emergency medical admission during the study period, and emergency medical admissions other than the index admission (defined as the chronologically first admission during the 4.5-year study period) were excluded. This was because not restricting analysis to index admissions would have meant that any deprivation gradients in readmission rates would have been confounded by deprivation gradients in index admissions, as previously described[11]. This is an important difference of the methodology used in this study in relation to the way the relevant performance indicators are presently calculated, including "all" (as opposed to index/first only) admissions in the denominator[12].

An emergency medical re-admission was defined as the first subsequent emergency medical admission during a follow-up period of either 28-days, or 3, 6 and 12 months respectively, following a first (index) emergency medical admission that led to a live discharge, and through the use of a single patient identifier. Observations were censored at the end of the chosen follow-up periods (as above) or at the time of intervening death unrelated to readmission to the study hospital. The latter was ascertained by data-linking to the Stockport Health Authority Public Health Mortality File produced by the Office for National Statistics.

### Measurement and definitions

Index admission data were originally available on: sex; age; length of stay of index admission; International Classification of Diseases (ICD)-10 coded primary diagnosis; number (up to four) of coded co-morbidities; patient post code and admission method (referral by Accidents and Emergency Department, General Practitioner or other). Information on primary diagnosis was aggregated into five categorical groups comprising chronic obstructive pulmonary disease / asthma (ICD codes J44.0–45.9 respectively), heart failure (I50.0–50.9), acute coronary syndrome (I20.0 [unstable angina] and I21.0–9 [acute MI]), stroke (I60.0–I67.0) and all other conditions (all other codes). Length of stay was divided into quartiles (<2, 2–5, 6–11 and >11 days). Deprivation status was subsequently ascribed with an area-based measure, using the 1991 Census enumeration district (ED) of patient's post-code, and by the use of Townsend multiple deprivation index score. Four deprivation groups were defined, using quartiles of the range of the Townsend scores between Stockport EDs.

### Analysis

Kaplan-Meier readmission-free curves at 28 days, and 3, 6 and 12 months were constructed for each of the following variables: *sex*, *age group*, *diagnostic group *(defined as above), *admission method*, *number of coded co-morbidities *(0–4), *length of stay group *(quartile) and *deprivation group *(quartile). Statistical significance for each of the above variables was assessed by the log rank test. A series of proportional hazards models with follow-up at 28 days, 3, 6 and 12 months were subsequently constructed to examine the adjusted hazard (risk) ratio of emergency medical readmission. Each model included all variables found to be significant in the uni-variable analysis at the 0.05 probability level. The proportional hazards assumption was tested using the Schoenfeld residuals as per the *stphtest *command in STATA. This showed that a time varying co-variate should be included in all four models, in relation to the *length of stay *variable (i.e. that an interaction term between *length of stay *and time of follow-up should be included), and this was hence included in the models.

Additionally, for the number of coded co-morbidities and deprivation group variables, a test for trend was performed, entering the actual values as continuous variables. In this context, the test for trend value indicates the proportional change in the risk of readmission associated with one unit change in the exposure variable (i.e. number of coded co-morbidities, Townsend deprivation score).

## Results

There were 21,118 index emergency medical admissions corresponding to an equal number of patients leading to a live discharge during the study period, but primary diagnosis information was only available for 20,209 index emergency medical admissions (Table 1). Cases without diagnostic information were excluded from further analysis.

**Table 1:** Basic characteristics of index admissions in study participants (n = 20,209)

**Variable**	**Category**	**n**	**%**
**Sex**	Male	9397	46.5
	Female	10812	53.5
**Age group**	<60	8094	40.1
	60–74	5526	27.3
	75+	6589	32.6
**Diagnostic group**	Acute coronary syndrome	4283	21.2
	COPD/asthma	1594	7.9
	Heart failure	587	2.9
	Stroke	575	2.8
	All other diagnoses	13170	65.2
**Length of Stay**	<2	5666	28.0
	2–5	5184	25.7
	6–11	5174	25.6
	>11	4185	20.7
**Deprivation Group**	Affluent	5057	25.0
	2	5003	24.8
	3	5113	25.3
	Deprived	5015	24.8
	Unknown	21	0.1
**Admission method**	A&E	12604	62.4
	GP referral	7113	35.2
	Other	492	2.4
**No of co-morbidities**	0	2963	14.7
	1	3796	18.8
	2	3790	18.8
	3	8801	43.5
	4	859	4.3

### Uni-variable analysis

**Figure 1:**
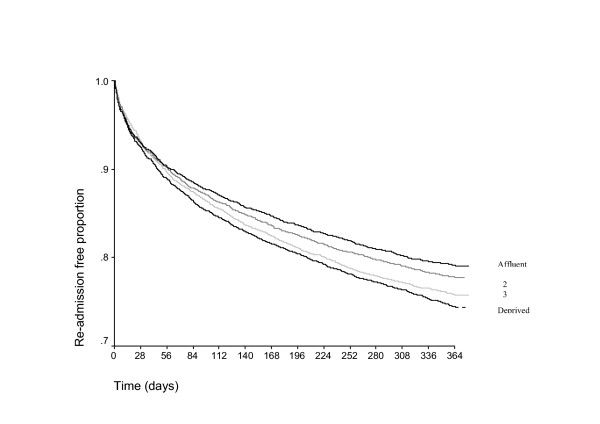
Kaplan-Meier readmission-free curves by deprivation group.

The proportion of patients readmitted at 28 days and 3, 6, 12 months progressively increased from 7.2% at 28-days to 23.3% at 12 months respectively (Table 2). Male sex, older age group, length of stay, higher number of coded co-morbidities and any primary diagnosis other than the "all other diagnoses" category were significantly associated with higher readmission rates independently of duration of follow-up (Table 2). Higher deprivation status was significantly associated with increased readmission rates in follow-up periods longer than three months, but not at 28 days (Table 2 and Figure 1). Admission method was not significantly associated with deprivation risk.

**Table 2:** Proportion of patients readmitted (%) by patient subgroup and different periods of follow-up (log rank test p values from relevant Kaplan-Meier readmission-free curves)

		**28 days**	p*	**3 months**	p*	**6 months**	p*	**12 months**	p*
**Sex**	Men	7.7	0.017	13.5	0.009	18.3	0.003	24.1	0.01
	Women	6.8		12.4		16.8		22.7	

**Age group**	<60	5.4	<0.001	8.8	<0.001	11.8	<0.001	15.8	<0.001
	60–74	8.2		15.1		19.7		26.4	
	>75	8.5		15.8		22.3		29.6	

**Admission method**	A&E	6.9	0.07	12.7	0.2	17.4	0.79	23.3	0.93
	GP referral	7.7		13.4		17.7		23.4	
	Other	7.8		11.6		17.6		23.9	

**Length of Stay**	<2	5.9	<0.001	8.6	<0.001	11.2	<0.001	14.7	<0.001
	2–5	6.5		10.9		15.5		21.4	
	6–11	8.0		15.6		21.2		28.0	
	>11	8.8		17.4		22.3		31.0	

**Number of coded co-morbidities**	None	6.1	<0.001	9.6	<0.001	13.2	<0.001	17.3	<0.001
	1	6.0		9.8		13.3		17.5	
	2	7.0		12.3		16.4		22.2	
	3	8.3		15.8		21.6		28.9	
	4	7.3		14.3		18.7		24.4	

**Diagnosis**	ACS	7.2	<0.001	13.0	<0.001	17.2	<0.001	22.3	<0.001
	COPD/Asthma	8.7		15.7		22.1		30.1	
	Heart Failure	11.1		22.7		31.3		37.5	
	Stroke	4.5		9.6		14.6		23.0	
	All other	7.0		12.3		16.6		22.3	

**Deprivation**	Affluent	7.1	0.44	11.8	0.002	15.8	<0.001	21.0	<0.001
	2	7.2		12.5		17.0		22.2	
	3	6.9		13.0		18.1		24.2	
	Deprived	7.7		14.3		19.2		25.9	
	
***Total***		***7.2***		***12.9***		***17.5***		***23.3***	

*Log Rank test

A&E: Accident and Emergency, GP: General Practitioner, ACS: Acute Coronary Syndrome, COPD: Chronic Obstructive Pulmonary Disease

### Multi-variable analysis

Male sex, older age group, and primary diagnosis of heart failure and chronic obstructive pulmonary disease/asthma were significantly associated with increased readmission risk, independently of length of follow-up (Table 3). With the "all other diagnoses" as the reference category, primary diagnosis of acute coronary syndrome was associated with a significantly increased risk of readmission at 3 and 6 months, but not at 28 days or 12 months. Independently of length of follow-up and all other variables, primary diagnosis of stroke was significantly associated with reduced readmissions risk compared with the "all other diagnoses" category as reference.

**Table 3:** Hazard ratios (HR) by variable and follow-up length, with associated 95% confidence intervals

**Variables in the Equation**	**28 days HR (95% CI)**		**3 months HR (95% CI)**		**6 months HR (95% CI)**		**12 months HR (95% CI)**	
**Female**	-		-		-		-	
								
Male	1.17**	*(1.06 – 1.30)*	1.14***	*(1.06 – 1.23)*	1.15***	*(1.08 – 1.23)*	1.13***	*(1.07 – 1.20)*
**Age <60**	-		-		-		-	
								
Age 60–74	1.41***	*(1.23 – 1.62)*	1.47***	*(1.33 – 1.64)*	1.44***	*(1.32 – 1.58)*	1.46***	*(1.35 – 1.58)*
Age >75	1.45***	*(1.26 – 1.67)*	1.45***	*(1.30 – 1.62)*	1.56***	*(1.42 – 1.71)*	1.57***	*(1.45 – 1.70)*
**Affluent**	-		-		-		-	
								
2	1.01	*(0.87 – 1.17)*	1.05	*(0.94 – 1.17)*	1.07	*(0.97 – 1.18)*	1.07	*(0.98 – 1.16)*
3	0.98	*(0.84 – 1.13)*	1.09	*(0.98 – 1.22)*	1.15**	*(1.05 – 1.27)*	1.17***	*(1.08 – 1.27)*
Deprived	1.09	*(0.94 – 1.26)*	1.21**	*(1.08 – 1.35)*	1.21***	*(1.10 – 1.33)*	1.25***	*(1.16 – 1.36)*
*Test for trend (Deprivation Index)*^	1.02	*(0.98 – 1.07)*	1.06***	*(1.03 – 1.10)*	1.07***	*(1.04 – 1.10)*	1.08***	*(1.05 – 1.11)*
**All other diagnoses**	-		-		-		-	
Heart Failure	1.32*	*(1.02 – 1.70)*	1.43***	*(1.19 – 1.71)*	1.47***	*(1.26 – 1.71)*	1.33***	*(1.16 – 1.53)*
COPD/asthma	1.25*	*(1.04 – 1.50)*	1.23**	*(1.08 – 1.41)*	1.29***	*(1.15 – 1.45)*	1.34***	*(1.21 – 1.48)*
ACS	1.12	*(0.97 – 1.28)*	1.15**	*(1.04 – 1.27)*	1.12*	*(1.02 – 1.22)*	1.07	*(0.99 – 1.15)*
Stroke	0.54**	*(0.37 – 0.81)*	0.57***	*(0.44 – 0.75)*	0.65***	*(0.52 – 0.81)*	0.76**	*(0.63 – 0.90)*
**Without co-morbidity**	-		-		-		-	
								
1 co-morbidity	1.02	*(0.83 – 1.25)*	1.04	*(0.89 – 1.23)*	1.05	*(0.91 – 1.20)*	1.10	*(0.97 – 1.24)*
2 co-morbidities	1.13	*(0.93 – 1.38)*	1.21*	*(1.03 – 1.41)*	1.19*	*(1.04 – 1.36)*	1.29***	*(1.15 – 1.45)*
3 co-morbidities	1.25*	*(1.04 – 1.49)*	1.39***	*(1.21 – 1.60)*	1.41***	*(1.25 – 1.59)*	1.54***	*(1.38 – 1.71)*
4 co-morbidities	1.26	*(0.93 – 1.69)*	1.46**	*(1.17 – 1.82)*	1.42***	*(1.18 – 1.73)*	1.49***	*(1.26 – 1.76)*
*Test for trend (number of com.)*^	1.08**	*(1.03 – 1.14)*	1.11***	*(1.08 – 1.17)*	1.13***	*(1.09 – 1.17)*	1.15***	*(1.12 – 1.18)*
**A&E referral**	-		-		-		-	
								
GP referral	1.09	*(0.98 – 1.22)*	1.00	*(0.92 – 1.09)*	0.96	*(0.89 – 1.03)*	0.93*	*(0.88 – 0.99)*
Other referral	1.17	*(0.85 – 1.60)*	0.84	*(0.64 – 1.09)*	0.91	*(0.74 – 1.13)*	0.94	*(0.78 – 1.13)*
**<2 days LoS**	-		-		-		-	
								
2–5 days LoS	0.54***	*(0.42 – 0.69)*	0.82*	*(0.69 – 0.99)*	0.92	*(0.79 – 1.08)*	1.06	*(0.93 – 1.21)*
6–11 days LoS	0.52***	*(0.41 – 0.66)*	0.89	*(0.74 – 1.05)*	1.14	*(0.98 – 1.32)*	1.31***	*(1.15 – 1.49)*
>11 days LoS	0.54***	*(0.41 – 0.70)*	0.95	*(0.79 – 1.14)*	1.23**	*(1.06 – 1.44)*	1.42***	*(1.25 – 1.62)*
<2 days LoS * Time^^	-		-		-		-	
								
2–5 days LoS * Time^^	1.07***	(1.05 – 1.09	1.01***	(1.01 – 1.02)	1.01***	(1.00 – 1.01)	1.002***	(1.001 – 1.003)
6–11 days LoS * Time^^	1.08***	(1.06 – 1.11	1.02***	(1.01 – 1.02)	1.01***	(1.00 – 1.01)	1.002***	(1.001 – 1.003)
>11 days LoS * Time^^	1.09***	(1.06 – 1.11	1.02***	(1.02 – 1.02)	1.01***	(1.00 – 1.01)	1.002**	(1.001 – 1.002)

*: p < 0.05, **: p < 0.01, ***: p < 0.001

^ : Denotes the proportion change in probability of outcome associated with one unit change in continuous variable (e.g. Townsend deprivation score index, number of co-morbidities)

^^ In days

HR: Hazard Ratio, COPD: Chronic obstructive airways disease, ACS: Acute coronary syndrome, LoS: Length of stay, A&E: Accident and Emergency, GP: General Practitioner.

More than two coded co-morbidities were associated with higher readmission risk only in follow-up periods of more than three months duration. However test for trend indicated a strong and significant positive effect of the number of coded co-morbidities independently of follow-up length.

Higher deprivation status was independently associated with higher readmission risk at 3, 6 and 12 months, but did not significantly influence readmission risk short term (at 28 days). Test for trend confirmed the strong and statistically significant effect of deprivation at 3–12 months but there was no effect at 28 days.

Admission method via a GP referral was significantly associated with a lower readmission risk at one year, but not at any other time intervals.

Length of stay of the index admission influences readmission risk differently, depending on the length of follow-up as there was a highly significant interaction between length of stay group and time of follow-up (see Additional file 1). Taking into account the relevant time varying co-variate, shorter length of stay is associated with higher readmission risk at discharge and immediately afterwards, but with lower readmission risk thereafter.

## Discussion

The findings indicate that in the study hospital about a quarter of patients with an index emergency medical admission will be readmitted in the same hospital during the subsequent year. Male sex and older age were strongly and independently associated with higher readmission risk, along with diagnosis of heart failure and chronic obstructive pulmonary disease or asthma. Effective measures to reduce readmission rates for patients suffering from these two conditions in particular are available[13,14] but not always used[13,15,16]. Improving the availability of effective treatments for these two conditions could contribute greatly to the management of demand for emergency care in general. The present study, at the local health economy level, has helped support the decision making process that allocated increased resources to the management of these two conditions by the expansion or creation of relevant specialist services.

As originally hypothesised, patient deprivation status exerted a significant independent effect on the risk of emergency medical readmission at 3–12 months of follow-up, with more deprived patients having had a higher readmission risk. There are several theoretical reasons why deprived patients may be at higher readmission risk, including: disease factors, such as greater disease severity in deprived patients[17]; patient factors, such as poor adherence to treatment and advice because of educational or behavioural reasons; and health and social care factors, such as differentials in the type and quality of primary care in particular, in a way analogous to the "inverse care law", originally describing differentials in access, rather than quality, of care[18]. A clearer understanding of the exact mechanisms responsible for deprivation group gradients through further research is necessary for future policy measures aiming at reducing such gradients.

Although this is a single-centre study, the results may also have implications for the way current and future NHS performance indicators relating to readmission rates are both constructed and interpreted. NHS hospitals serving pre-dominantly deprived populations might in principle be disadvantaged if indicators are not adjusted for the impact of deprivation on case-mix. Although this study showed no significant effect of deprivation status on readmission risk at 28-days, which is the follow-up period currently used by the performance indicators[1,2], care should be taken when interpreting this "negative" finding. Firstly, this analysis included in the denominator only index (as opposed to "all") admissions, in contrast to the technical specification of the performance indicators[12]. Because more deprived patients have higher rates of index emergency medical admissions[11], including all index admission in the calculation will accentuate any deprivation differences in readmission risk, and the performance indicators as they are currently calculated may for this reason be misleading. Previous analysis of the same dataset including "all" admissions provides empirical evidence that this is true[19]. Secondly, it is possible that a true effect of deprivation status on index readmission rates at 28-days also exists but it was not detected by our study due to its single-centre nature, or insufficient sample size. A larger study, ideally using data from more than one hospital may be warranted.

The Department of Health includes performance indicators in the calculations of award of "three-star" status, which in turn is the "gateway" to "Foundation" status"[20]. Standardising, or otherwise adjusting, for patient deprivation is feasible using the HES data, as this study indicates. Standardisation of readmission indicators for patient deprivation status would be prudent. This would ensure that NHS organisations serving deprived communities would not be unfairly "punished" for poor performance because of factors outside their control. It will also increase the perception of validity of the indicators. Unlike information on disease severity, which is difficult to measure accurately for most medical conditions, information about patient socioeconomic status using area-based (ecological) deprivation measures is relatively easy to obtain, using patient postcodes, routinely included in the Hospital Episodes Statistics dataset.

All studies using administrative data are sensitive to the quality of routine data collection. The validity of HES data in relation to age, sex, length of stay, admission method, and area of residence is generally good, but misclassification errors may occur in relation to diagnostic codes and the extent to which co-morbidities are recorded and coded[21]. Currently the degree of miscoding in our data is uncertain, but an audit of 200 cases in the study hospital has shown diagnostic inaccuracy to be in the order of 7.5%, comparable with levels quoted in the published literature[22]. Misclassification of primary diagnosis might be assumed to have occurred non-differentially between patients of different deprivation groups, and is so it would have diminished rather than exaggerated any association observed in this study, including the observed effect of deprivation. Misclassification error may have also resulted by the use of ecological measures of socioeconomic status (ecological fallacy). Again, this would reduce the effect size, if one exists. Therefore the effect of deprivation status on readmissions risk reported in our study may be an under-estimate of a true association.

A limitation of the study is that, besides the very large sample size, the findings are based on one single hospital in an urban English setting, and in principle the results are not generalisable. Similarly, the study was not population-based, so readmissions that may have occurred to other hospitals (either because of where patients happened to be taken if fallen acutely ill, or due to migration) were not ascertained. In theory such readmissions may have occurred at a differential rate between different deprivation groups. However this factor is unlikely to have biased the results in any considerable way for two reasons. First, the emergency (as opposed to elective) nature of the studied condition (emergency medical admission) makes it unlikely that either patients or doctors exercise an important degree of choice on which hospital a patient is admitted or readmitted, independently of patient deprivation status. Second, due to local geography and service configuration, 85% of the total medical admissions in Stockport residents occur at Stockport NHS (unpublished data). Similarly, by the nature of the hospital-centred nature of the study, admissions to private hospitals could not have been accounted. However, most admissions to private hospitals are for elective surgical procedures (rather than emergency medical reasons) for which we believe this is unlikely to have introduced considerable degree of bias. Lastly it is worth remembering that current NHS performance indicators for hospital Trusts are not population-based.

## Conclusions

Our study suggests that there is an important potential for both managing emergency demand and improving individual patient experience by focusing on the effective management of heart failure and chronic obstructive pulmonary disease. Although there is a similar potential by reducing differentials in readmission risk between deprivation groups, more research is required in order to understand reasons for such differentials in order to inform relevant policy measures. In the mean time, standardisation or other adjustment of hospital readmission indicators for patient socio-economic status in the future would be prudent. Failure to do so may disadvantage hospitals serving primarily deprived communities.

## Competing interests

The author(s) declare that they have no competing interests.

## Authors' contributions

The study was conceived and designed by GL and GC. GL, DH and IG analysed data. All authors contributed in the interpretation of findings and in the writing of the paper. GL and GC are guarantors.

## Pre-publication history

The pre-publication history for this paper can be accessed here:



## Supplementary Material

Additional File 1 Length of stay group and readmission free Kaplan-Meier curves (0–28 days). This file demonstrates that during follow-up of 0–28 days, length of stay is not proportional to readmission risk, reason for which a time varying co-variate was included in the Cox regression model (see main article Text). Click here for file
