# Factors influencing emergency medical readmission risk in a UK district general hospital: A prospective study

**DOI:** 10.1186/1471-227X-5-4

**Published:** 2005-07-18

**Authors:** Georgios Lyratzopoulos, Daniel Havely, Islay Gemmell, Gary A Cook

**Affiliations:** 1 Directorate of Clinical Services and Public Health, Norfolk, Suffolk and Cambridgeshire Strategic Health Authority, Fulbourn, UK; 2 Evidence for Population Health Unit, University of Manchester, Manchester, UK; 3 Department of Epidemiology, Stockport NHS Trust, Stockport, UK

## 

We recently received a query from a reader of our article [1], about the ICD-10-based definitions used to define the "chronic obstructive pulmonary disease / asthma" group of index admissions. Prompted by the query, we detected an error in the way the definition of this group, as well as the "acute coronary syndrome" group are described in the printed version of our article, which we wish to correct.

The printed version of the article describes chronic obstructive pulmonary disease / asthma as composed of ICD-10 codes J44.0-45.9. This should have read: **J40.0-J46 (i.e. diagnostic codes relating to Chronic Obstructive Pulmonary Disease, chronic bronchitis, emphysema and asthma), as well as J12.0-J18 (i.e. diagnostic codes relating to pneumonia). **We have included asthma and pneumonia in the definition of this group because, based on experience, we felt that in adults over 50 who are admitted to hospital a primary diagnosis of either asthma or pneumonia is very likely to either represent miscoding of COPD for (asthma or pneumonia), and/or an exacerbation episode of underlying Chronic Obstructive Pulmonary Disease. We include the exact composition of this group, by diagnostic code in Table 1.

**Table 1:** ICD-10 coding composition of the "chronic obstructive pulmonary disease / asthma" group of index admissions used in the study.

ICD-10 code	Description	Number of index admissions	%
**J13X**	**Pneumonia due to Streptococcus pneumoniae**	18	0.6
**J14X**	**Pneumonia due to Haemophilus influenzae**	2	0.1
**J151**	**Bacterial pneumonia, not elsewhere classified**	3	0.1
**J154**	**Bacterial pneumonia, not elsewhere classified**	4	0.1
**J157**	**Bacterial pneumonia, not elsewhere classified**	10	0.3
**J180**	**Pneumonia, organism unspecified**	53	1.7
**J181**	**Pneumonia, organism unspecified**	450	14.8
**J182**	**Pneumonia, organism unspecified**	1	0.0
**J188**	**Pneumonia, organism unspecified**	1	0.0
**J189**	**Pneumonia, organism unspecified**	201	6.6
**J42X**	**Unspecified chronic bronchitis**	2	0.1
**J439**	**Emphysema**	61	2.0
**J440**	**Other chronic obstructive pulmonary disease**	771	25.3
**J441**	**Other chronic obstructive pulmonary disease**	632	20.7
**J448**	**Other chronic obstructive pulmonary disease**	11	0.4
**J449**	**Other chronic obstructive pulmonary disease**	157	5.2
**J450**	**Asthma**	1	0.0
**J459**	**Asthma**	657	21.6
**J46X**	**Status asthmaticus**	12	0.4
**Total**		3047	100

Similarly, in the printed version of the article the group of "acute coronary syndrome" is defined as I20.0 and I21.0-9. This should have read: **I20.0-9 (i.e. angina), I21.0-9 (acute MI) and R07.0-9 (i.e. "non-specific pain in throat or chest"). **Again we have decided to include non-specific chest pain along with angina and MI, due to potential for miscoding and/or true co-morbidity (of non-specific chest pain with acute ischaemic heart disease presentations). The exact composition of codes used for the "acute coronary syndrome" group is included in Table 2.

**Table 2:** ICD-10 coding composition of the "acute coronary syndrome" group of index admissions used in the study.

ICD-10 code	Description	Number of index admissions	%
**I200**	**Unstable angina**	367	8.6
**I201**	**Angina pectoris with documented spasm**	2	0.0
**I208**	**Other forms of angina pectoris**	3	0.1
**I209**	**Angina pectoris, unspecified**	721	16.8
**I210**	**Acute transmural myocardial infarction of anterior wall**	285	6.7
**I211**	**Acute transmural myocardial infarction of inferior wall**	388	9.1
**I212**	**Acute transmural myocardial infarction of other sites**	40	0.9
**I214**	**Acute subendocardial myocardial infarction**	90	2.1
**I219**	**Acute myocardial infarction, unspecified**	363	8.5
**R072**	**Precordial pain**	2	0.0
**R073**	**Other chest pain**	626	14.6
**R074**	**Chest pain, unspecified**	1396	32.6
Total		4283	

## Pre-publication history

The pre-publication history for this paper can be accessed here:

http://www.biomedcentral.com/1471-227X/5/4/prepub
