# Supplementary material for: Factors influencing emergency medical readmission risk in a UK district general hospital: A prospective study
Source: BMC Emerg Med. 2005 Jan 21;5:1. doi: 10.1186/1471-227X-5-1 (PMC548275; doi:10.1186/1471-227X-5-1)
Supplement: Additional File 1 — Length of stay group and readmission free Kaplan-Meier curves (0–28 days). This file demonstrates that during follow-up of 0–28 days, length of stay is not proportional to readmission risk, reason for which a time varying co-variate was included in the Cox regression model (see main article Text). [file 1471-227X-5-1-S1.doc]

Figure. Length of stay group and readmission free Kaplan-Meier curves 0-28 days.
